# Preliminary reflections of CAMHS in COVID-19 lockdown

**DOI:** 10.1017/ipm.2020.33

**Published:** 2020-05-14

**Authors:** Sunnhild Bertz, Oluwatobi Olatoye, Susan O’Hanrahan, Nazuk Zaidi

**Affiliations:** CAMHS, HSE, Unit 6, Quinn Road Business Park, Ennis, Co Clare, Republic of Ireland; CAMHS, HSE, Ennis, Co Clare, Republic of Ireland

Child and Adolescent Mental Health Service (CAMHS) in Co Clare consists of two teams (East and West sectors) serving a catchment area of 100,000 population. This piece reflects on and examines various questions/issues which have arisen for our CAMHS in Co Clare, in the first 4 weeks of the nationwide COVID-19 pandemic lockdown. Given the considerable uncertainty in current times, it is intended to highlight some preliminary views, with the aim of re-visiting issues in post-pandemic times. It describes some of the changes observed in the service since the restrictions imposed in mid-March and how these might inform service provision in CAMHS going forward.

The emerging literature relating to mental health in the COVID-19 pandemic suggests that psychological reactions to pandemics which include maladaptive behaviours, emotional distress and defensive responses are all features which are in clear evidence during the current COVID-19 pandemic (Cullen *et al*. 2020). Since the enforced reduction of the CAMHS in Co Clare in response to the COVID-19 crisis, these responses have, however, not yet come to our attention.

These tentative observations have prompted reflections on the human response to adversity and the purpose, role and function of a CAMHS.

While routine out-patient appointments took place over the phone following the nationwide lockdown imposed in mid-March 2020, in only 25 cases was it deemed necessary for the patient and family to attend in person. Twenty-one of these cases were seen in clinic and four were seen at their homes.

Reflecting on the potential contributory role of the human response to crisis has raised the following suppositions/questions. Are patients and their parents in fact far more resilient than we might give them credit for? Do we all have an innate resilience in times of crisis, something which was described by authors such as Rodriguez-Llanes *et al*. (2013) in examining psychological resilience in disaster settings?

Has there been a shift in the hierarchy of needs and priorities on the part of patients and, perhaps more likely, their parents? This fall-off in patient numbers has of course also been observed in other areas, for example, Emergency Department presentations for non-COVID-19 issues. Is it that parents (and patients) are in ‘fight or flight’ mode, with more basic survival instincts emerging, where in an era of acridity, physical and economic survival take precedence? Is it as if we are ‘at war’ and that the ‘war effort’ is built on a promotion (and indeed a necessity) of greater self-reliance, with an increased focus on individual households as the critical unit in attempts to stem the pandemic?

Anecdotal evidence gleaned from phone consultations during the early stages of the crisis (first 4 weeks) suggests that in many instances, parents have indicated that their children/teenagers have been less anxious, with more stable mood and improved sleep since the closure of schools in March 2020. Has the enforced lockdown shown us the capacity of families to adjust positively (at least in the short term) to re-focus on the family unit and utilisation of latent skills? Could the enforced school holidays have led to an improvement in child mental health?

Has accessibility to the CAMHS (due to the reluctance of patients to present to their General Practitioner and Emergency Departments) skewed our observations relating to presentations? Or is this to be expected as we know that mental health slips further down the hierarchy of needs during times of crises? Importantly, could this fall-off in presentations be viewed as merely ‘the calm before the storm’ as we have not yet observed the predicted surge in mental health presentations?

Reflections on the purpose, role and function of the CAMHS have led us to consider the following issues. The number and type of presentations (and its implications to CAMHS post-pandemic) will undoubtedly be heavily influenced by the wider and uncertain socio-economic fallout of the disease, coupled with revised family dynamics, a return to school, exams and everyday pressures. We do not yet know what shape this landscape will take. Thus, a key challenge will be how to prepare for an unknown health, financial and social context and as uncertainty unfolds in the world at large, our modus operandi of offering containment, reassurance and managing uncertainty is challenged.

Our apparent success in managing clinic appointments by telephone may indicate the potential for developing aspects of the service through the medium of telepsychiatry. This time of transition may well be an opportunity to develop this further. This will need to take into account training around patient confidentiality and the skill of tuning into non-verbal aspects of interpersonal communication.

These questions have prompted us to consider what are the non-negotiables in the CAMHS? The pared-back service over the last 4 weeks has emphasised three domains, namely risk assessments, the assessment of severe mental illness and prescribing. Further consideration is required around critical domains for CAMHS which is likely to be operating under increasingly stringent conditions (reduced public finances, post-pandemic demands and expectations).

Does this crisis provide the opportunity to revisit the essence of a CAMHS and re-evaluate the Operational Guidelines for CAMHS with a view to enhance the delivery of services? The key importance of continuing to evaluate how youth mental health services are delivered within the context of changing societal environments and needs has been highlighted (Alexander & Lyne, 2019). This is true now more than ever. We view the inevitability of the current uncertainty as an opportunity to adapt and improve our current service provision. It is a time to reflect on how the service can better move forward in an evolving landscape. The speciality of CAMHS can now re-evaluate its core identity and harness the knowledge acquired throughout these challenging times.
